# Critical-depth Raman spectroscopy enables home-use non-invasive glucose monitoring

**DOI:** 10.1371/journal.pone.0197134

**Published:** 2018-05-11

**Authors:** Signe M. Lundsgaard-Nielsen, Anders Pors, Stefan O. Banke, Jan E. Henriksen, Dietrich K. Hepp, Anders Weber

**Affiliations:** 1 RSP Systems, Odense, Denmark; 2 Department of Endocrinology, Odense University Hospital, Odense, Denmark; 3 Endocrinology and Diabetology, Munich, Germany; Harvard Medical School, UNITED STATES

## Abstract

One of the most ambitious endeavors in the field of diabetes technology is non-invasive glucose sensing. In the past decades, a number of different technologies have been assessed, but none of these have found its entry into general clinical use. We report on the development of a table-top confocal Raman spectrometer that was used in the home of patients with diabetes and operated for extended periods of time unsupervised and without recalibration. The system is based on measurement of glucose levels at a ‘critical depth’ in the skin, specifically in the interstitial fluid located below the stratum corneum but above the underlying adipose tissue layer. The region chosen for routine glucose measurements was the base of the thumb (the thenar). In a small clinical study, 35 patients with diabetes analyzed their interstitial fluid glucose for a period of 60 days using the new critical-depth Raman (CD-Raman) method and levels were correlated to reference capillary blood glucose values using a standard finger-stick and test strip product. The calibration of the CD-Raman system was stable for > 10 days. Measurement performance for glucose levels present at, or below, a depth of ~250μm below the skin surface was comparable to that reported for currently available invasive continuous glucose monitors. In summary, using the CD-Raman technology we have demonstrated the first successful use of a non-invasive glucose monitor in the home.

## Introduction

Diabetes mellitus, in its different forms, is affecting an increasing number of individuals and placing undue strain on national health care budgets. Estimates (from 2015) state that 415 million people worldwide suffer from diabetes whilst this number is predicted to increase to 642 million by 2040 [1]. For control of treatment the self-monitoring of blood glucose is recommended, which is usually performed with an invasive finger-prick method. In Type 1 diabetes patients dosing of insulin is frequently based on 4 to 6 blood glucose determinations per day. For these reasons, it has been a long term goal to develop truly non-invasive techniques to measure the blood glucose levels of diabetes patients. The current clinical trend favors indwelling Clarke-type (i.e., electrochemical) sensors that allow for continuous glucose monitoring in a minimally-invasive way [2,3]. However, a skin puncture is still required, with associated discomfort for the user and increased risk of infection; whilst these sensors also suffer from a biocompatibility issue that limits their life to a few weeks [4].

For many decades it has been a goal to develop non-invasive techniques to measure the blood glucose levels of diabetes patients but practical solutions for general use have so far not been developed. The majority of approaches have been based on optical measurement of glucose in tissues such as the skin [5,6,7]. Amongst these the spectroscopic techniques, such as fluorescence, absorbance and Raman have attracted considerable attention. Despite the fact that inelastic Raman scattering is a weak process and thus results in a poor signal the associated high chemical specificity, minimal interference from tissue water content and only a modest fluorescence background render the technique one of the most promising candidates for non-invasive glucose monitoring [8]. Since the first feasibility study of measuring blood glucose with near-infrared Raman spectroscopy in 1997 [9] several groups have substantiated this claim by quantitative measurements of glucose levels in vivo [10–14]. These reports may be considered proof-of-concept only in the sense that all measurements were performed in a controlled environment whilst the predictive capabilities of the calibration model were assessed by cross-validation only.

In this paper we describe the design and development of a table-top, confocal near-infrared Raman instrument for intermittent glucose determination. The instrument uses a new principle of critical-depth Raman where measurements are taken from interstitial fluid within a defined region of the skin. It is worth noting that in contrast to previous work that also utilizes a confocal setup to probe in the living part of the skin [10,14], the current work is the first of its kind to systematically study the relation between probing depth and prospective performance of the Raman-based glucometer, thus allowing us to define a critical depth from which the Raman signal should be acquired. Moreover, we report on the first uncontrolled study where the system has been used by patients with diabetes in their home over a period of 60 days to obtain accurate blood glucose measurements.

## Materials and methods

### Instrumentation

**Fig 1:**
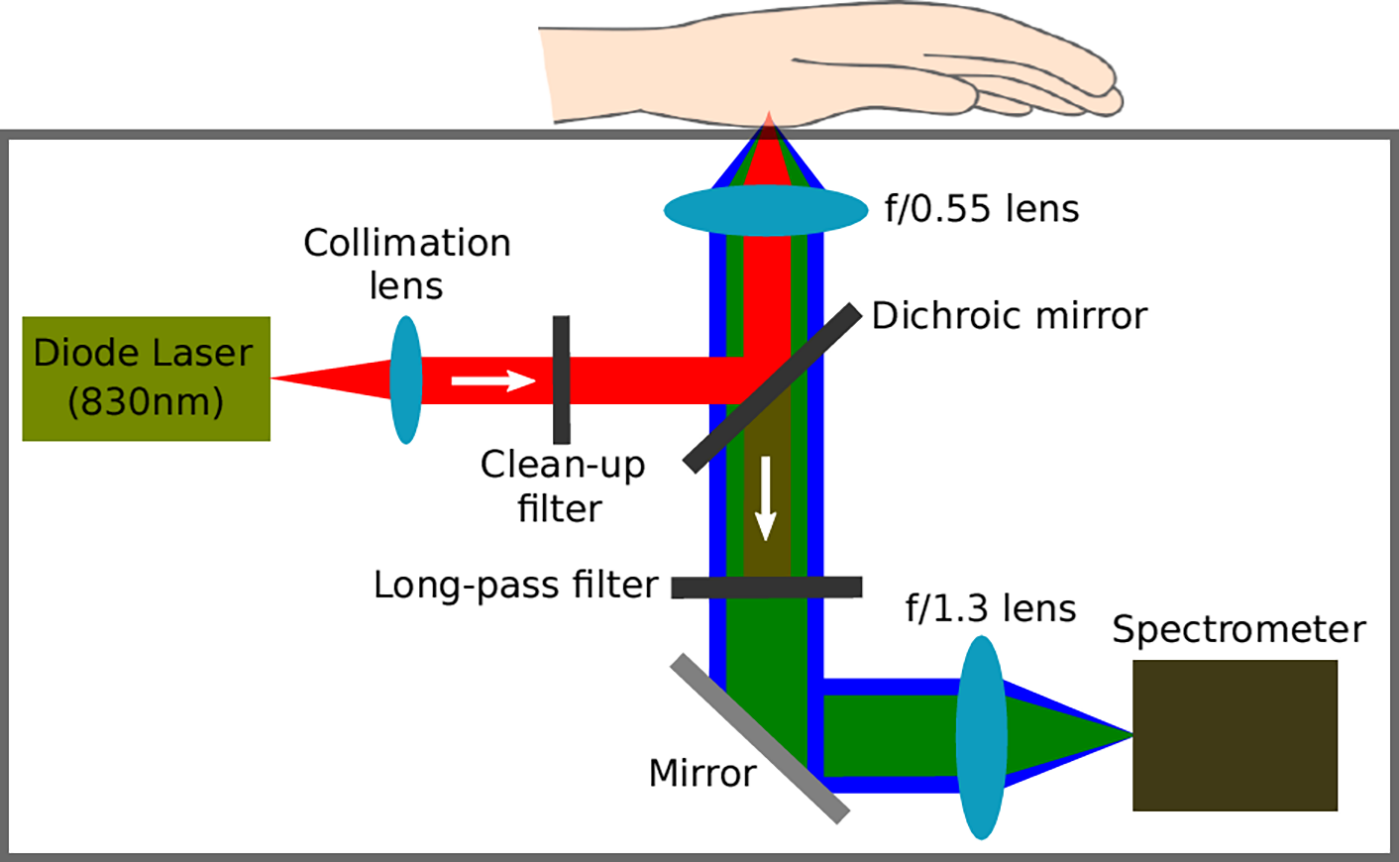
Schematic presentation of portable Raman spectrometer. The output from the diode laser (300mW continuous power) is first collimated, unwanted spectral side lobes and fluorescence are filtered, then redirected by the dichroic mirror and finally focused (by the f/0.55 lens) just below the skin surface. Whilst both the intense reflected/scattered light, fluorescence, and generated Raman photons are collected by the f/0.55 lens, the dichroic mirror and the long-pass filter ensure that only the latter two contributions to the spectrum are focused (by the f/1.3 lens) on the entrance slit of the spectrometer.

The spectra were recorded using a custom-built confocal Raman setup of external dimensions 475mm (l) x 212mm (h) x 361mm (w). Fig 1 displays a schematic diagram of the internal components.

The laser light is generated by a continuous-wave diode laser that emits at a wavelength of 830nm with a power of 300mW (I0830MU0350MF-NL from Innovative Photonic Solutions, New Jersey, USA). Before the laser light interacts with the skin, it is first collimated and sent through a clean-up filter to remove unwanted spectral side lobes and background fluorescence. Then, the excitation light is redirected by a dichroic mirror and strongly focused onto the thenar (base of the thumb) by the probe lens (f-number = 0.55). The thenar is placed on a 500μm thick magnesium fluoride window that is the only opening in the otherwise opaque cover. The interaction of the excitation light with the skin generates Rayleigh scattered light, as well as broadband fluorescence background, and molecule-specific Raman photons; these are all collected and collimated by the probe lens. The Rayleigh light, however, is suppressed by the dichroic mirror and the long-pass filter, only allowing wavelengths above 850nm to reach the spectrometer slit. Before the light enters the spectrometer, it is focused onto the 50μm x 100μm entrance slit that also functions as the pinhole in our confocal setup. The spectrometer has a f-number of 1.3 with a spectral resolution of 9cm^-1^ in the measurement range of 300-1800cm^-1^ (custom-configured WP830 spectrometer from Wasatch Photonics, North Carolina, USA). The charge-coupled detector (1024x64 pixels) within the spectrometer is temperature stabilized at 15°C. It should be noted that all filters and mirrors are from Semrock (New York, USA), while lenses are custom-made in order to minimize optical aberrations and maximize the throughput.

**Fig 2:**
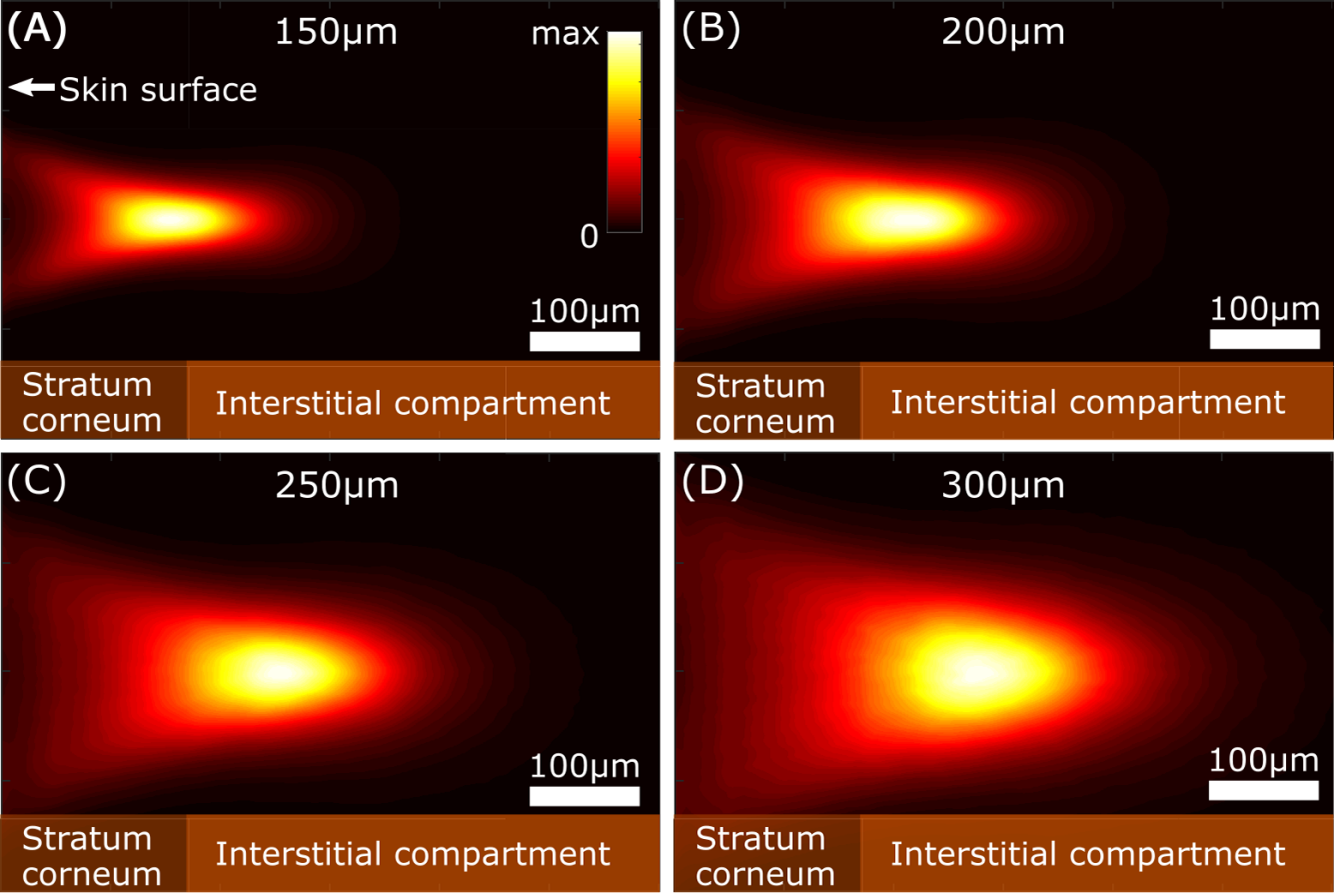
Simulation of signal acquisition density distribution. Panels (A)-(D) show the simulated density distribution of the generated Raman signal in the skin for collection depths of 150, 200, 250, and 300μm, respectively. The skin is modelled as a turbid medium featuring both bulk and surface scattering.

The depth selectivity in our device is a consequence of the confocal design, wherein the focus/collection depth (the ‘critical depth’, or CD) below the skin surface can be controlled by changing the distance between the probe lens and the reference plane of the hand (see Fig 1). In this study, we used a distance range of approx. 150 to 300μm from the reference plane at the skin surface to the focus point in the interstitial fluid. An illustration of the expected Raman signal density distribution in the skin (calculated as the square of the incident flux) is shown in Fig 2 for CDs of 150, 200, 250, and 300μm.

The simulation was obtained by modelling the Raman signal in Zemax OpticStudio 16.5 (Zemax LLC, Seattle, WA), where the surface roughness and inhomogeneity of the skin are addressed by including Trowbridge-Reitz surface scattering [15] and Henyey-Greenstein bulk scattering [16]. It is important to note that the exact thickness and optical properties of the various skin layers vary between people, so Fig 2 should only be considered a general illustration of the light-skin interaction of our optical setup and the influence of varying the CD. For further details of the skin model, we refer to S1 Appendix. The simulations suggest that roughness and turbidity in the skin leads to an increased volume from which Raman photons are collected as the CD increases. Moreover, with the average measured stratum corneum (SC) of the thenar encompassing the initial 166μm of the skin (see Protocol), it is clear that a larger fraction of the Raman signal must originate from the interstitial compartment of the skin with increasing CD. As the dynamic glucose signal (which reflects blood levels) is expected from this component of the skin only, the simulations indicate that a confocal Raman-based CD glucose monitor should focus and collect light from below the SC layer.

### Study protocol

Raman spectra were recorded from the thenar of 41 patients with diabetes aged between 20 to 60 years recruited from Odense University Hospital, Department of Endocrinology, Denmark. For all patients the thickness of the SC at the thenar was measured using an optical coherence tomography system (Callisto, Thorlabs Inc. New Jersey, USA). The average SC thickness was 166 +/- 40μm (x +/- SD). Six patients with SC thickness greater than 206μm were excluded from the subsequent data analysis. The remaining cohort of 35 patients comprised 14 males and 21 females, of which 32 had Type 1 and 3 had Type 2 diabetes; the CD of the Raman device was fixed for each patient according to the selection criteria described in Results and was within the range 140 to 320μm.

The patients were equipped with our table-top CD-Raman device (as described in Fig 1) and instructed in how to use the device at home. Each patient enrolled in the study could freely choose the 30 days of analysis within a period of 60 days, where each analysis day included four measurement sessions (chosen appropriately over the day); which also included reference glucose determinations conducted in capillary blood with HemoCue 201 RT (HemoCue AB, Ängelholm, Sweden). Each session consisted of two subsessions with acquisition times of 2 and 1 minute respectively and the resulting Raman spectrum was a weighting of the two spectra according to the acquisition times. It should be noted that the total acquisition time of 3 minutes is chosen based on performance, whereas the influence of the split has not been investigated in this study. Between the two subsessions the hand was removed from the device to ensure that the prospective performance was not critically dependent on achieving a precise measurement site on the thenar. As it is of paramount importance in real-life usage that the calibration model can encompass such lateral variations in measurement site on the thenar, we have made no attempt to quantify (or restrict) the scale of displacement between subsessions and sessions. It should be noted that accidental movement of the hand during acquisition is not detected on-the-fly, but it always results in error-prone spectra that can be removed during data analysis. The study was approved by the National Danish Committee on Health Research Ethics and the Danish Medicines Agency, and there is written consent from all the participants. The study protocol is publicly available at ClinicalTrials.gov, identifier NCT03368209.

### Data analysis

The analysis of the measured Raman spectra and the correlation to blood glucose levels was centralized in Matlab R2017a (MathWorks, Natick, Massachusetts, USA) using the PLS Toolbox (Eigenvector Research Inc., Manson, Washington, USA).

As a first step, we excluded saturated spectral data and error-prone spectra due to misplacement of hand or interference from ambient light. Secondly, measurement sessions where the blood glucose reference was reported to exceed 30 mmol/L were excluded to conform with the consensus error grid [17] and the measurement range of the reference glucometer Hemocue 201RT. No other data exclusion criteria were used in this study, and the remaining Raman spectra used in the following data analysis can be found in S1 Dataset with S2 Appendix giving an accompanying description of the different data fields.

After data capture the spectra were first smoothed by a 15-point Savitsky-Golay 1st order algorithm, then corrected for varying intensities and backgrounds using 6^th^ order EMSC (extended multiplicative scatter correction [18]), and finally mean-centered. The reference blood glucose concentrations were mean-centered prior to analysis. We note that both the number of points used for smoothing and the EMSC order are rather high, but the values are chosen based on overall optimal predictive performance of the calibration models. We foresee that future implementation of outlier detection techniques may reduce these parameter values.

For the quantitative evaluation of our CD-Raman glucometer the data was divided into a calibration set (initial 25 days of analysis) and an independent validation set (final 5 days of analysis), with the correlation between Raman spectra and blood glucose concentrations being determined through partial least squares (PLS) regression. The choice of 25 calibration days is a consequence of the PLS models improving up to this point. Based on the calibration set we calculated a PLS model for each patient, where the optimal number of latent variables (LVs) was determined from simultaneous minimization of the root-mean-square error (RMSE) of calibration and 10-fold cross-validation (CV, contiguous blocks). For further validation (both cross- and independent validation) of the PLS models, we evaluated the fraction of samples in the zones of a consensus error grid, mean average relative difference (MARD), slope and intercept for a best linear fit between reference and predicted glucose concentrations, correlation coefficient R^2^ and RMSE. It is worth noting that the temporal behaviour and range of blood glucose values are very dependent on the patient with diabetes. Some of the patients were very well-regulated with glucose values in the range of 4–10 mmol/L, with occasional hyperglycaemic episodes, whilst others exhibited a broader glucose range of 1–30 mmol/L with hypo-, eu-, and hyperglycaemic periods observed. The differing behaviour amongst the patients is challenging for quantifying the performance using a single validation parameter. To overcome this problem, we have developed a composite performance parameter called Inter-Subject Unified Performance (ISUP):

$$\mathrm{ISUP}=(A+B-MARD)\cdot slope\cdot R^{2}\cdot 1[\mathrm{mM}]/Y_{\mathrm{median}},$$ (1)

where *A* and *B* are the percentage distribution of samples in zone A and B in the consensus error grid, *MARD* is the calculated MARD in percentage, *slope* is the slope of the best linear fit in the consensus error grid, *R*^*2*^ is the correlation coefficient and *Y*_median_ is the median reference glucose value. The composite performance parameter is evaluated on cross- or independent validated results and is designed to weight the presence of samples in zone A and B, together with a low MARD, a high slope and a high correlation coefficient. Furthermore, the ISUP takes into account that the glucose levels in some patients are generally well-regulated by also weighting the median of blood glucose concentrations.

## Results

**Fig 3:**
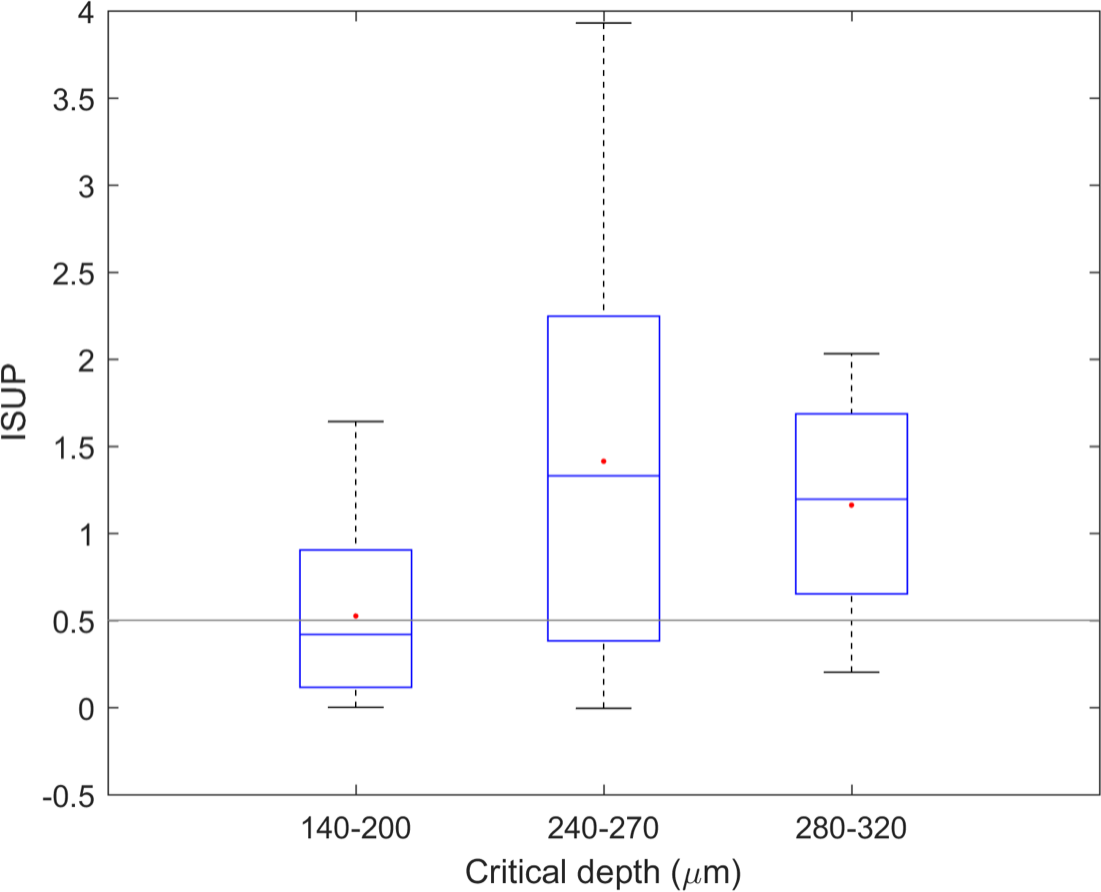
Box plot of performance versus collection depth. 35 patients were grouped into three depth ranges as indicated: 140–200 um, n = 14; 240–270 um, n = 12; 280–320 um, n = 9. The box shows upper and lower quartile and median. The threshold of 0.5 was supported by the CV results and the grading of glucose similarity for the regression vector, above this value the performance was satisfactory.

The cohort of 35 patients was assigned to 3 groups for determination of analytical performance at depths of 140–200 μm, 240–270 μm, and 280–320 μm below the reference plane. Data quality was expressed by the ISUP parameter as described in Methods (Data Analysis). A depth limit of 320 μm was chosen because of the gradual deterioration of the signal below this threshold. The boxplot in Fig 3 shows the median ISUP for the three patient groups, where a score of 0.5 was set as the threshold between acceptable and poor performance. The choice of threshold was supported by the cross-validated results for each patient, together with the regression vector of the calibration model and a comparison with the Raman spectrum from a reference glucose solution. The performance data in Fig 3 indicates a positive correlation between the CD and ISUP. An optimal collection depth of 250–300 μm was chosen for the subsequent study; which is consistent with the simulations shown in Fig 2. It is reasonable therefore to assume that the probe mainly collected the Raman signal from the interstitial fluid compartment below the SC.

**Fig 4:**
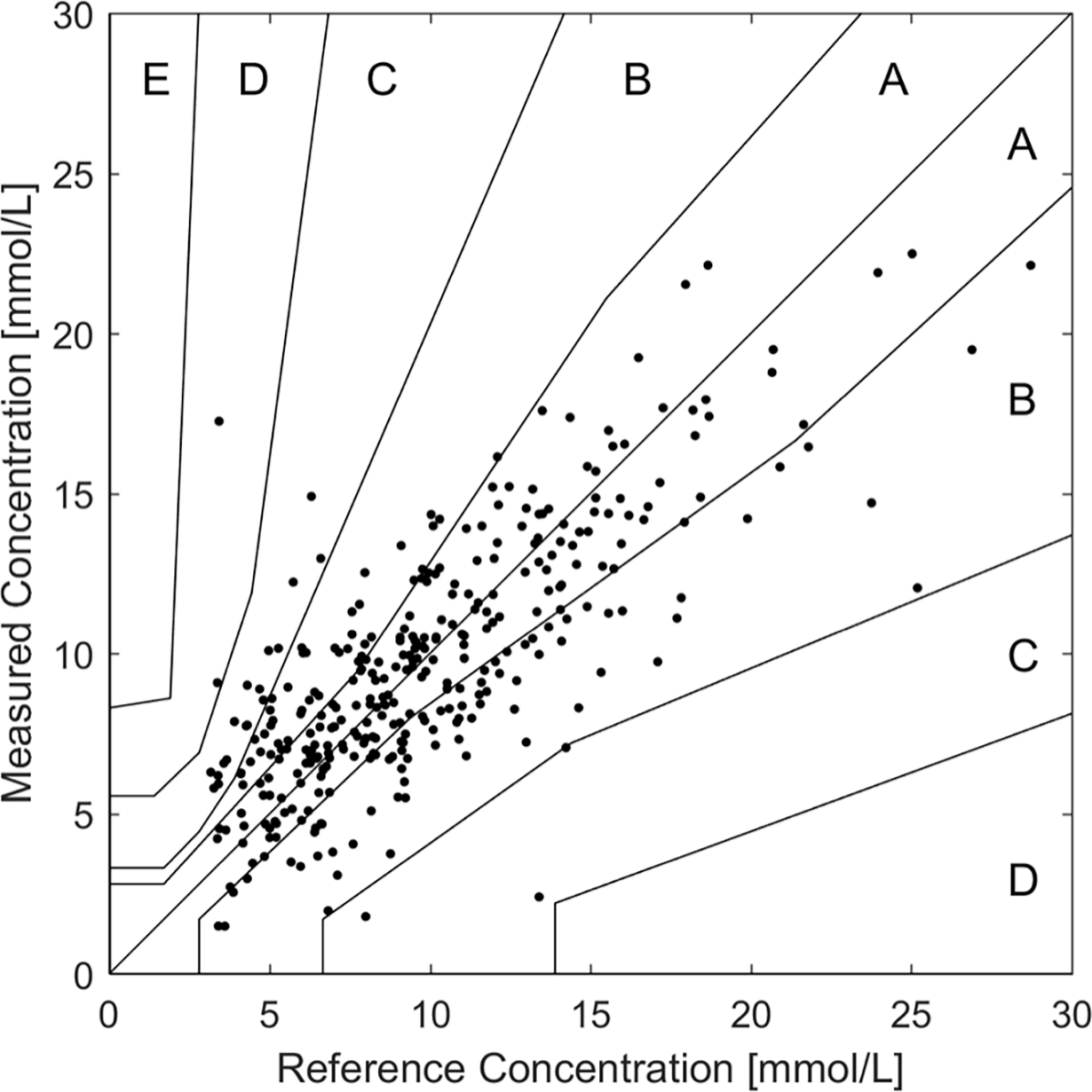
Independent validation of the last five days of analysis. The measured glucose concentrations are plotted as a function of reference values in a consensus error grid. The measurement critical depth was 240–320 um and the ISUP was greater than 0.5.

In 15 patients, featuring ISUP above 0.5 and CD in the range of 240–320 μm, five days of data were validated independently with reference to their capillary glucose values as described in Methods. On the consensus error grid shown in Fig 4, the independent validations are represented with a slope of 0.69, a MARD of 25.8% with 93% of predictions in the areas A + B. A comparison of cross- and independent validation is shown in Table 1. As the performance of the two validation methods is nearly identical this shows the suitability of the cross-validation method and the predictive capabilities of the generated PLS models.

**Table 1:** Performance of cross-validated (CV) and independent validations (IV).

	CV	IV
**MARD (%)**	27.3	25.8
**Slope**	0.65	0.69
**A (%)**	54.1	58.3
**B (%)**	39.8	35.2
**C (%)**	5.4	5.9
**D (%)**	0.7	0.6
**E (%)**	0	0
**Number of samples**	1530	338
**Patients**	15	15

**Fig 5:**
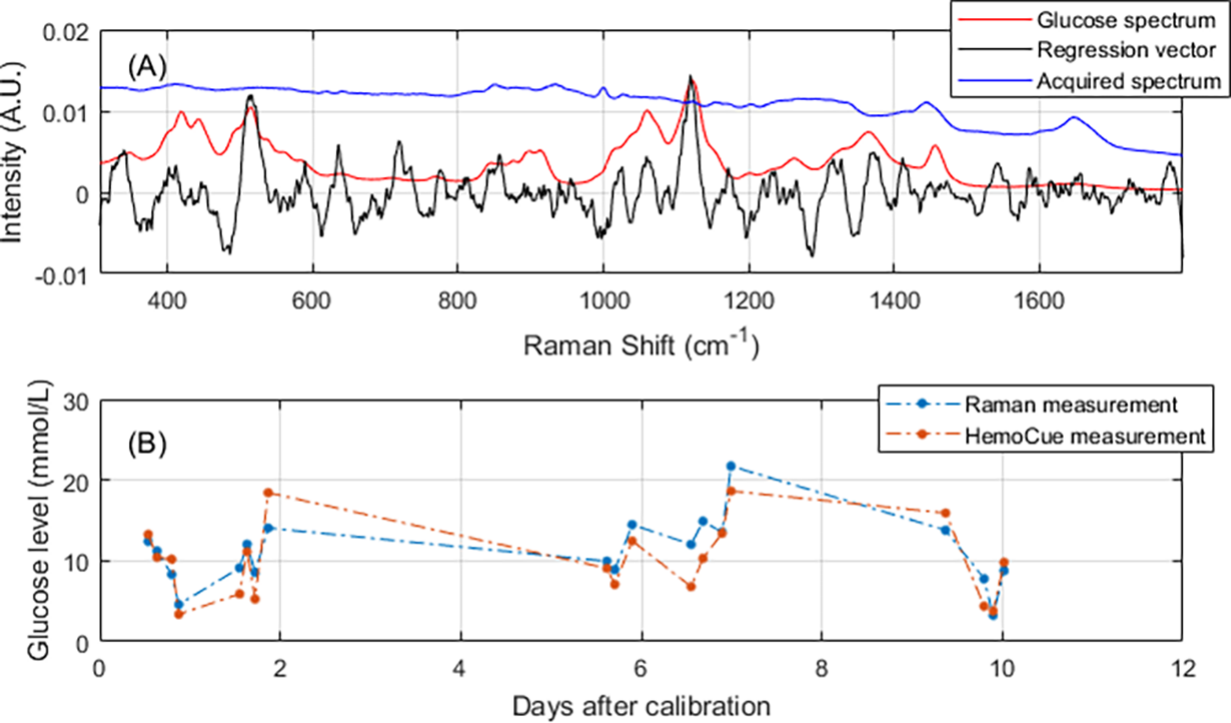
Raman spectrum and independent predictions for an average-performing patient. (A) Average Raman spectrum from the thenar region (blue), Raman spectrum of glucose in an aqueous solution (red), and regression vector of PLS calibration model (black). (B) Plot of the independently predicted glucose concentration and the reference value as a function of time from the last day of calibration. The dashed lines are for guidance only.

Fig 5 shows the results obtained from one patient (patient ID: 15), while the remaining results for the other 14 patients can be found in S1 Fig and S2 Fig. The representative patient was an average-performing individual with CD of 250μm, ISUP of 1.9 and MARD from independent validation of 27.6%. Fig 5A shows a comparison between the average Raman spectrum from the thenar, the spectrum of a glucose reference solution and the regression vector of the PLS calibration model. It is seen that the presence of glucose is not immediately evident in the raw thenar spectrum, which consists of a broad fluorescence background overlaid by mainly skin-specific Raman peaks. In contrast, the regression vector shows clear spectral signatures of glucose, with significant peaks at the glucose-specific Raman bands at 514 cm^-1^ and 1123 cm^-1^. Fig 5B shows the measurements from the independent validation set as a function of time from the last day of calibration. For this patient the last five days of analysis extended over a period of ten days in total and it is clear that the predictive performance did not deteriorate during this time; thus, calibration was stable for at least 10 days.

## Discussion

In the present paper we report on the development of a table-top confocal, critical-depth Raman spectrometer that can be used in the home of patients with diabetes and operated for extended periods of time (at least 60 days) without supervision. In a small clinical study, we have shown that the resulting Raman spectra obtained from the thenar of 35 patients with diabetes can be correlated with blood glucose values; demonstrating that, with spectral data collection at a depth of approx. 250μm the analytical capabilities of CD-Raman were unambiguously verified with a calibration that is maintained for at least 10 days. The existence of a critical measurement depth is in line with the broadly accepted view that it is necessary to probe beyond the SC to measure dynamic glucose levels. Probing beyond depths of ~300μm has not been investigated in this work due to the decreasing Raman signal for increasing probing depths (a consequence of skin turbidity) and the risk of an unwanted signal from the underlying adipose tissue. We also note that the calibration may be valid for more than 10 days, but the currently acquired data doesn’t support a claim exceeding this time frame.

Selecting data obtained with a critical depth of greater than 240μm and ISUP larger than 0.5 gave an independent validation of 93% for readings in zone A and B of the consensus error grid and an overall MARD of 25.8%; a performance that is close to current invasive continuous monitoring devices that typically show a MARD between10-30% and 90–98% reading in zone A+B [2,3].

In general, there are several factors which can influence the accuracy by which the CD-Raman system can measure interstitial glucose concentration. One such factor is the time lag of changes in glucose concentration between the capillary and interstitial compartments that may vary in relation to glucose diffusion kinetics. It is generally accepted that the interstitial glucose concentration is delayed in comparison with the blood value during transient situations [19,20]. The time lag has been reported to be up to 45 minutes, depending on measurement site and technique used [21]. Lag time associated with the IF compartment, just below the SC, is most likely close to 5 minutes [22]. Finally, a considerable bias can be introduced by the capillary reference method [23,24].

As we have shown, calibration is based on an individual PLS model for each patient. For the further development of the CD-Raman system it will be necessary to establish a global calibration strategy that can deal with the individual characteristics of patients, including differences in the optical properties of the skin. We are currently investigating the possibilities for applying machine learning techniques, categorization and non-linear regression models. [12,25].

In conclusion, this small clinical study using CD-Raman to measure interstitial glucose suggests that a paradigm shift in diabetes care is now within reach. We are currently designing extended clinical trials that will be used to further evaluate the success of this new spectroscopic approach. In the future we will be miniaturizing the Raman setup to reduce the footprint of the device and eventually allow wearable devices for non-invasive continuous monitoring of blood glucose levels, as discussed previously by M. S. Wróbel [8].

## Supporting information

S1 Fig Raman spectra and regression vectors for 14 patients featuring an ISUP above 0.5 and a critical depth in the range of 240–320 μm. (TIF)

S2 Fig Independent prediction of glucose concentration and the reference value as a function of time from the last day of calibration for 14 patients, all featuring an ISUP above 0.5 and a critical depth in the range of 240–320 μm. (TIF)

S1 Appendix Detailed description of the skin model used in simulations. (PDF)

S2 Appendix Explanation of data fields in S1 Dataset. (PDF)

S1 Dataset Unprocessed Raman spectra and related information for the 35 patients involved in the data analysis. (MAT)
