# Supplementary figures and images for: Critical-depth Raman spectroscopy enables home-use non-invasive glucose monitoring
Source: PLoS One. 2018 May 11;13(5):e0197134. doi: 10.1371/journal.pone.0197134 (PMC5947912; doi:10.1371/journal.pone.0197134)

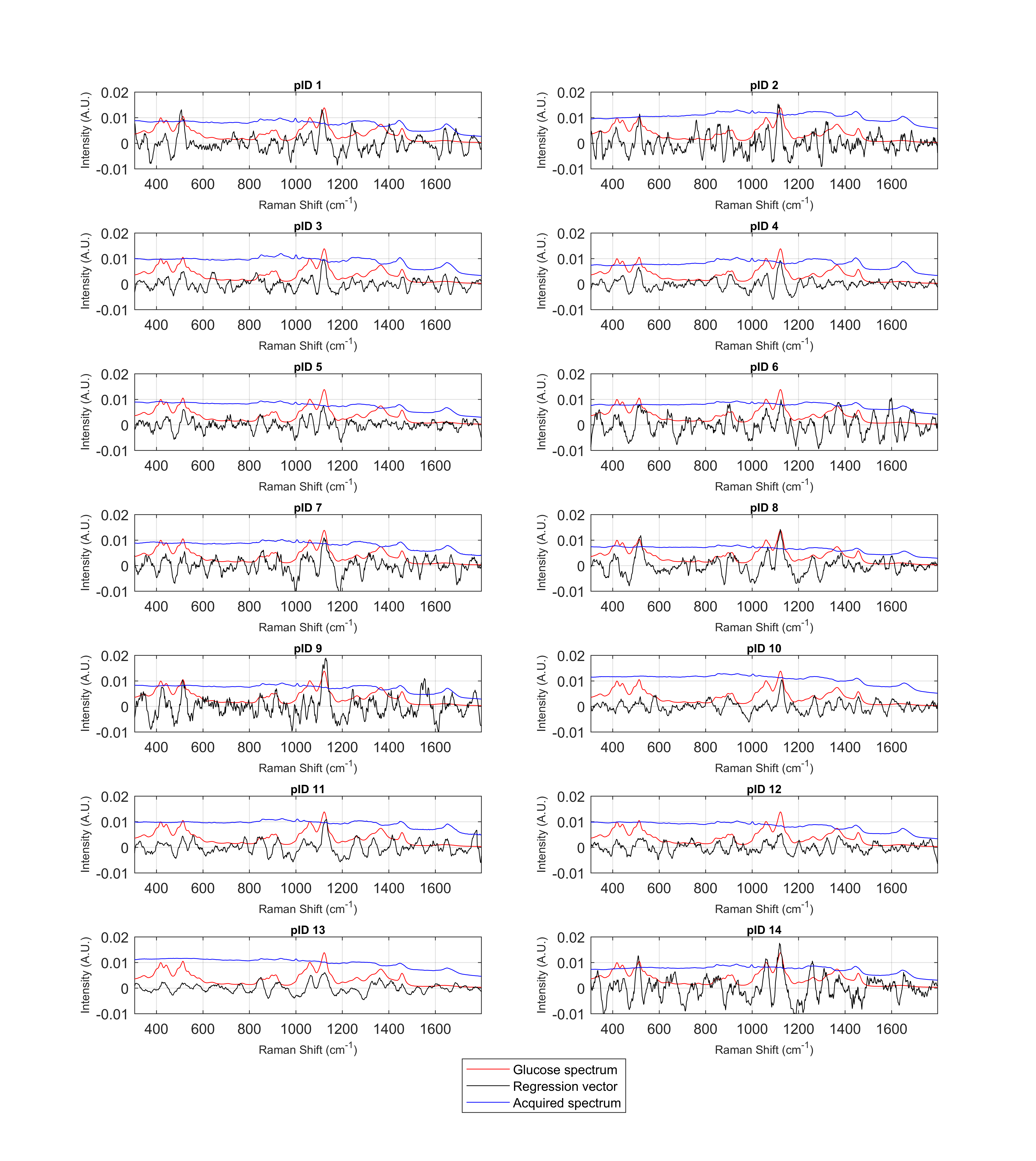

Supplement: S1 Fig — (TIF) [file pone.0197134.s001.tif]

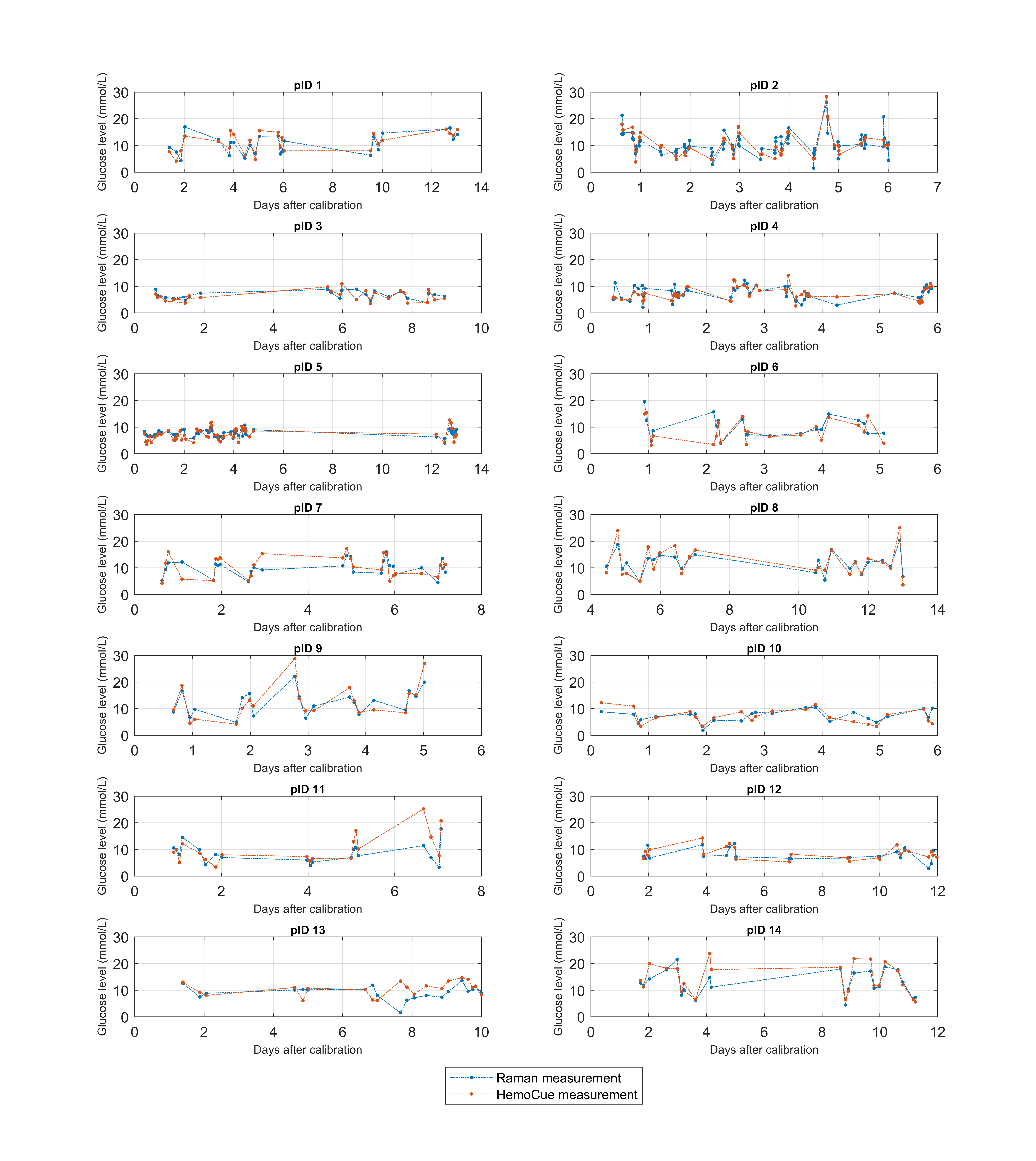

Supplement: S2 Fig — (TIF) [file pone.0197134.s002.tif]
