# Supplementary material for: Critical-depth Raman spectroscopy enables home-use non-invasive glucose monitoring
Source: PLoS One. 2018 May 11;13(5):e0197134. doi: 10.1371/journal.pone.0197134 (PMC5947912; doi:10.1371/journal.pone.0197134)
Supplement: S1 Appendix — (PDF) [file pone.0197134.s003.pdf]

Supporting Information (S1 Appendix) for

Critical-depth Raman spectroscopy enables home-use non-invasive glucose monitoring

Signe M. U. Christensen[1], Anders Pors[1], Stefan O. Banke[1], Jan E. Henriksen[2], Dietrich K. Hepp[3], Anders Weber[1*]

[1] RSP Systems, Odense S, Denmark

[2] Department of Endocrinology, Odense University Hospital, Odense, Denmark

[3] Endocrinology and Diabetology, Munich, Germany

* Corresponding author

E-mail: andersw@rspsystems.com

The Skin Model

The very complex interaction of light with the (on the micrometer scale) rough and inhomogeneous skin is modelled in Zemax OpticStudio 16.5 by assuming the skin to consist of 5 homogeneous layers that have different optical properties, including a scattering mean free path that represents the scattering of the light due to the inhomogeneity of the skin. The skin parameters utilized in this work can be seen in S1 Table 1.

The first row in S1 Table 1 indicates the simple two-layer skin model illustrated in the main manuscript, where the interstitial compartment represents skin layers from which a dynamic glucose signal can be extracted. The second row in the table represents the 5-layer skin model used during simulations, where the stratum corneum is divided into two layers, with the first layer being strongly scattering. The thicknesses of the different layers are derived from OCT measurements on the thenar of the patients included in the clinical trial. Similarly, the scattering mean free paths, representing the average distance a photon travels before it undergoes a scattering event, are derived from the OCT measurements. The Albedo describes the probability that a photon undergoes scattering rather than absorption, and the values close to one are a result of using the near-infrared wavelength of 830nm where the absorption in the skin is weak. The anisotropy factor is a parameter in the Henyey-Greenstein bulk scattering model that describes whether light is scattered in any preferential direction. An anisotropy factor close to one means that light is dominantly scattered in the forward direction. Finally, it should be noted that the rough surface of the skin is modelled using the Trowbridge-Reitz scattering model with a roughness parameter of 0.3. The roughness parameter has been determined by fitting the scattering model to BRDF (bidirectional reflectance distribution function) measurements on the palm.

	Stratum Corneum		Interstitial Compartment		
	Stratum Corneum 1	Stratum Corneum 2	Living Epidermis	Papillary Dermis	Reticular Dermis
Thickness (µm)	50	116	50	135	(Assumed thick)
Refractive index	1.52	1.47	1.42	1.42	1.38
Scattering mean free path (µm)	29	200	430	91	160
Albedo	0.999	0.999	0.992	0.992	0.990
Anisotropy factor	0.95	0.95	0.86	0.86	0.86

S1 Table 1. Optical properties of 5-layer skin model.
