# Supplementary material for: Critical-depth Raman spectroscopy enables home-use non-invasive glucose monitoring
Source: PLoS One. 2018 May 11;13(5):e0197134. doi: 10.1371/journal.pone.0197134 (PMC5947912; doi:10.1371/journal.pone.0197134)
Supplement: S2 Appendix — (PDF) [file pone.0197134.s004.pdf]

Supporting Information (S2 Appendix) for

# Critical-depth Raman spectroscopy enables home-use non-invasive glucose monitoring

Signe M. U. Christensen[1], Anders Pors[1], Stefan O. Banke[1], Jan E. Henriksen[2], Dietrich K. Hepp[3], Anders Weber[1*]

[1] RSP Systems, Odense S, Denmark

[2] Department of Endocrinology, Odense University Hospital, Odense, Denmark

[3] Endocrinology and Diabetology, Munich, Germany

* Corresponding author

E-mail: andersw@rspsystems.com

## Description of data fields in S1 Dataset

The unprocessed Raman spectra (and related information) that are acquired during the clinical trial and used for data analysis are collected in a .mat file in a struct named *Xfilt*.

The struct *Xfilt* contains the following information:

- Data: Unprocessed Raman spectra.
- ID: Information related to the individual Raman spectra:
    - Col. 1: Person no. (pID)
    - Col. 2: Analysis day
- NumDate: Time stamp as a decimal number, representing number of days from the 1$^{st}$ of January 2015.
- Settings: Information regarding device settings of collection depth (in µm).
- Skin: Information regarding the thickness (in µm) of stratum corneum on thenar.
- Yref: Reference glucose concentration (in mmol/L) from blood sample.
- Axis: Raman shifted axis ($cm^{-1}$).
